# Potentially preventable hospitalisations for physical health conditions in community mental health service users: a population-wide linkage study

**DOI:** 10.1017/S204579602100007X

**Published:** 2021-03-10

**Authors:** G. Sara, W. Chen, M. Large, P. Ramanuj, J. Curtis, F. McMillan, C.L. Mulder, D. Currow, P. Burgess

**Affiliations:** 1 InforMH, System Information and Analytics Branch, NSW Ministry of Health, Sydney, Australia; 2 Northern Clinical School, Sydney Medical School, University of Sydney, Sydney, Australia; 3 School of Psychiatry, University of NSW, Sydney, Australia; 4 Royal National Orthopaedic Hospital, London, England; 5 RAND Europe, London, England; 6 School of Nursing, Midwifery & Indigenous Health, Charles Sturt University, Wagga Wagga, Australia; 7 Epidemiological and Social Research Institute, Erasmus University, Rotterdam, Netherlands; 8 Cancer Institute NSW, Sydney, Australia; 9 School of Public Health, University of Queensland, Brisbane, Australia

**Keywords:** Comorbidity, Multimorbidity, Hospitalisation, Mental Disorders

## Abstract

**Aims:**

Mental health (MH) service users have increased prevalence of chronic physical conditions such as cardio-respiratory diseases and diabetes. Potentially Preventable Hospitalisations (PPH) for physical health conditions are an indicator of health service access, integration and effectiveness, and are elevated in long term studies of people with MH conditions. We aimed to examine whether PPH rates were elevated in MH service users over a 12-month follow-up period more suitable for routine health indicator reporting. We also examined whether MH service users had increased PPH rates at a younger age, potentially reflecting the younger onset of chronic physical conditions.

**Methods:**

A population-wide data linkage in New South Wales (NSW), Australia, population 7.8 million. PPH rates in 178 009 people using community MH services in 2016–2017 were compared to population rates. Primary outcomes were crude and age- and disadvantage-standardised annual PPH episode rate (episodes per 100 000 population), PPH day rate (hospital days per 100 000) and adjusted incidence rate ratios (AIRR).

**Results:**

MH service users had higher rates of PPH admission (AIRR 3.6, 95% CI 3.5–3.6) and a larger number of hospital days (AIRR 5.2, 95% CI 5.2–5.3) than other NSW residents due to increased likelihood of admission, more admissions per person and longer length of stay. Increases were greatest for vaccine-preventable conditions (AIRR 4.7, 95% CI 4.5–5.0), and chronic conditions (AIRR 3.7, 95% CI 3.6–3.7). The highest number of admissions and relative risks were for respiratory and metabolic conditions, including chronic obstructive airways disease (AIRR 5.8, 95% CI 5.5–6.0) and diabetic complications (AIRR 5.4, 95% CI 5.1–5.8). One-quarter of excess potentially preventable bed days in MH service users were due to vaccine-related conditions, including vaccine-preventable respiratory illness. Age-related increases in risk occurred earlier in MH service users, particularly for chronic and vaccine-preventable conditions. PPH rates in MH service users aged 20–29 were similar to population rates of people aged 60 and over. These substantial differences were not explained by socio-economic disadvantage.

**Conclusions:**

PPHs for physical health conditions are substantially increased in people with MH conditions. Short term (12-month) PPH rates may be a useful lead indicator of increased physical morbidity and less accessible, integrated or effective health care. High hospitalisation rates for vaccine-preventable respiratory infections and hepatitis underline the importance of vaccination in MH service users and suggests potential benefits of prioritising this group for COVID-19 vaccination.

## Introduction

Mental health (MH) conditions are associated with reduced life expectancy. Disadvantage, lifestyle, increased prevalence of chronic illness, medication side effects, reduced help-seeking and the accessibility and quality of health care all contribute to this mortality gap (Lawrence *et al*., 2010; Liu *et al*., 2017; Firth *et al*., 2019). Action on this problem requires complex health system changes across the spectrum of prevention, primary care and specialist mental and physical health services (Liu *et al*., 2017; Barber and Thornicroft, 2018; Firth *et al*., 2019).

Effective indicators are essential for health system change (Berwick *et al*., 2003). This study examines Potentially Preventable Hospitalisations (PPH) as a possible indicator of medical morbidity and health care in people with MH conditions. PPH, also called ‘Ambulatory Care Sensitive Conditions’, are widely used indicators of health system effectiveness (Ansari, 2007; Falster and Jorm, 2017; Kim *et al*., 2019). PPH are hospitalisations for specific conditions that are potentially avoidable through prevention and community care. PPH indicator definitions differ between countries, but typically include chronic conditions (such as chronic obstructive airways disease, angina, asthma or diabetes complications), acute conditions (such as convulsions, appendicitis, urinary tract infections or cellulitis), and some vaccine-preventable conditions (such as influenza) (Australian Commission on Safety and Quality in Health Care, 2017; Agency for Healthcare Research and Quality, 2018; Canadian Institute for Health Information, 2019; National Health Service Digital, 2019).

PPH may provide a useful indicator of excess medical morbidity in MH service users. They include many of the disorders contributing to morbidity and mortality in people with mental illness, including conditions related to smoking, lifestyle and metabolic health (Ansari, 2007; Falster *et al*., 2015). They reflect access to health care and integration between primary and specialist health services (Parchman and Culler, 1994; Basu *et al*., 2002; Gulliford, 2002; Gibson *et al*., 2013; Kim *et al*., 2019), both of which contribute to health disparities in people with MH conditions (Firth *et al*., 2019). They are routinely reported in many health systems (Agency for Healthcare Research and Quality, 2018) (Australian Commission on Safety and Quality in Health Care, 2017; Canadian Institute for Health Information, 2019; Ministry of Health, 2019; National Health Service Digital, 2019) and hence are a familiar and accepted measure of health inequalities for many policy-makers and clinicians.

Increased PPH rates have been reported in people with schizophrenia (Lin *et al*., 2011; Davydow *et al*., 2015; Kisely *et al*., 2015), substance use disorders (Mai *et al*., 2011) and bipolar disorder (Mai *et al*., 2011). These studies have examined PPH rates in cohorts with follow-up periods ranging from 5 years (Lin *et al*., 2011; Davydow *et al*., 2015; Kisely *et al*., 2015) to 16 years (Mai *et al*., 2011). However, if we aim to support health system improvement and assess change then more timely measures are needed. Long observation periods limit the potential of PPH as health system indicators, because policy-makers and clinicians may not see them as reflecting recent health system behaviour. Long observation periods also make indicators insensitive to change, because system improvements may not be reflected for many years.

The current study has two aims. First, to examine short-term (12-month) rates of PPH in MH service users, examining whether increased rates observed in longer-term follow-up studies are also observed over shorter time periods more suitable for routine indicator reporting. Second, to examine the relationship between age and the risk of PPH admission. People with MH conditions have a younger age at onset of risk factors such as obesity and conditions such as cardiovascular disease (De Hert *et al*., 2011). Therefore, we hypothesise that people with MH conditions will have an earlier onset of PPH as well as increased prevalence of PPH. To our knowledge, no study has examined this issue.

## Methods

### Study setting

Australian health services are primarily government-funded. Approximately 65% of MH service expenditure is by state and territory governments, who fund acute and emergency hospital care, acute community care and long-term community MH care for people with a severe or enduring illness. New South Wales (NSW) state government hospital and community services are provided through 15 geographically organised Local Health Districts and three Speciality Health Networks, which are responsible for physical care (hospital and some outpatient care) as well as MH care. State government-operated community MH services are organised around geographical catchment areas, are free of charge and can be accessed without referral by a General Practitioner (primary care physician). Private office-based primary and specialist care, private hospital care, and pharmaceuticals are funded or subsidised by the Australian Federal Government with a varying degree of consumer co-payment. Referral by a General Practitioner is required to access subsidised private specialist care. Private hospitals mainly provide non-emergency care (including voluntary MH care) for individuals opting in to private health insurance: in the study period, private hospitals provided 23% of total acute overnight hospital episodes in NSW and 27% of acute overnight hospital days (Australian Institute of Health and Welfare, 2018).

### Study design

An observational study was carried out to identify all PPH admissions over 12 months in the state of NSW, Australia, with population of 7.8 million. Dependent variables were the number of PPH admissions and PPH hospital days, expressed as rates per 100 000 population. Independent variables were (i) MH service user status, defined as any contact with state specialist MH services in the preceding 2 years, (ii) age and (iii) socio-economic status of person's area of residence. MH service user status was defined by linkage to state-wide community MH datasets.

### Study group: contact with specialised community MH services

The study group (MH group) were NSW residents aged 0–100 years who had clinical contact with any NSW government (public sector specialised) ambulatory MH service between 1 July 2015 and 30 June 2017. These included face-to-face, telephone or videoconference contacts in community MH centres, home visits, hospital outpatient clinics or emergency departments. Services to non-NSW residents, administrative contacts, case conferences and in-reach services to hospital inpatients were excluded. Age, sex and area of residence were defined at the index contact in the observation period. Primary care, private specialist care and non-government organisation contacts are not available in the dataset.

### Outcome: potentially preventable hospitalisations over 12 months

The main outcome was admission to any NSW public or private hospital in 1-year observation period between 1 July 2016 and 30 June 2017 with a primary or secondary diagnosis of a PPH, as defined using Australia's national specification (Australian Commission on Safety and Quality in Health Care, 2017). This definition includes 21 conditions organised in three groups: (i) acute conditions such as gastroenteritis or urinary tract infection where primary care should detect and treat most cases without the need for hospital care; (ii) chronic conditions such as asthma and diabetes, where effective ongoing disease management should minimise complications and prevent the need for hospitalisation, and; (iii) vaccine-preventable conditions such as rubella and tetanus, where the incidence of the disorder should be largely prevented by effective primary care (see Supplementary Table S1 for detailed specifications).

### Data linkage

The study is part of the NSW *Health Mental Health Living Longer* (MHLL) program, a data linkage examining premature mortality in MH service users. Data were linked by the NSW Centre for Health Record Linkage (CHeReL; www.cherel.org.au). Data sets and linkage methods are described elsewhere (Sara *et al*., 2019).

### Data analysis

Data assembly and standardisation were conducted in SAS Enterprise Guide v7.15., and SAS v9.4. Statistical estimates and comparisons were conducted Stata v15.

The number of PPH and hospital bed days in the observation period were measured for each person. Twelve-month PPH rates per 100 000 people were calculated separately for the MH group and non-MH groups. PPH episode and day rates were calculated for (i) any PPH, (ii) three PPH groups (chronic, acute and vaccine-preventable conditions) and (iii) 21 individual PPH conditions. For ease of interpretation, chronic conditions were further sub-grouped into cardiovascular, metabolic and respiratory conditions, and acute conditions into acute infections, seizures and other acute conditions. Episodes with multiple PPH diagnoses were counted separately for condition-specific rates but treated as a single episode when calculating overall PPH rates. Age-specific rates were calculated using 5-year bands defined by age on 30 June 2016. Differences in the proportion of people with any PPH were tested using a two-sample test of proportions. Differences in the number of PPH episodes per person and the average length of stay of PPH episodes were compared using Wilcoxon's rank-sum test.

Adjusted Incidence Rate Ratios (AIRRs) were calculated by direct standardisation to the NSW non-MH population distributions for age group and for the socioeconomic disadvantage index of the person's area of residence (expressed as a quintile). Disadvantage was measured using the Australian Bureau of Statistics Index of Relative Socioeconomic Disadvantage (IRSD) (Australian Bureau of Statistics, 2006), an index score calculated for each Australian geographical area by combining 17 census-derived variables related to income, government welfare support, education, home-ownership, employment, household structure and English language proficiency (for more information see Supplementary material Table S6). Confidence intervals (95%) for rates and AIRRs were estimated assuming a Poisson distribution.

Planned sensitivity analyses were conducted to examine the potential effects on findings of exclusion of (i) PPHs occurring prior to first MH contact in the observation period, (ii) admissions to private hospitals and (iii) PPH with associated MH care.

### Ethics approval

The study was approved by the NSW Population and Health Service Research Ethics Committee (HREC/17/CIPHS/48. CINSW Refs 2017/|HRE1105, 2019/UMB0208) and the Aboriginal Health and Medical Research Council of NSW (Ref 1564/19).

### Project governance

The study is overseen by (i) a Steering Committee which includes representation from peak organisations representing NSW health consumers, MH service users and MH carers, and (ii) an Aboriginal Advisory Committee which includes Aboriginal people with lived experience, community organisation, policy and research expertise.

## Findings

A total of 178 009 people had contact with NSW ambulatory MH services in the observation period. Half (50.1%) were male. MH service users were younger than the NSW population: most were aged between 15 and 44 (57% of MH service users, 41% of NSW population), see Supplementary Table s2. Diagnostic characteristics of our community cohort are described elsewhere: the most common specific diagnoses are affective disorders, psychotic disorders and other MH conditions including anxiety and adjustment disorders (Sara *et al*., 2019).

MH service users comprised 2.3% of the population but experienced 6.9% of PPH and 9.3% of PPH bed days (Table 1). MH service users were more likely to have had any PPH, multiple PPH during the study period, and a longer average length of stay for PPH episodes.

**Table 1.** Potentially Preventable Hospitalisations (PPH) in NSW residents in 2016–2017, comparing people with and without recent mental health (MH) service use

	MH service users		Other NSW residents	
	*n*	%	*n*	%
Population (*n*, % of NSW population)	178 009	2.3	7 561 265	97.7
PPH episodes (*n*, % of episodes)	11 635	6.9	157 671	93.1
PPH days (*n*, % of total days)	63 134	9.3	615 534	90.7
People with at least one PPH (*n*, % of group)	7877	4.4 ***	127 667	1.7
Episodes per person (*n*)	1.5 ***		1.2	
Average length of stay (days)	5.5 ***		3.7	

***p < 0.0001.

After adjustment for age and disadvantage, MH service users experienced 3.6 (95% CI 3.5–3.6) times the rate of PPH compared to other NSW residents (Table 2). AIRR were elevated by 3.3 (95% CI 3.2–3.4) for acute conditions, 3.7 (95% CI 3.6–3.7) for chronic conditions and 4.7 (95% CI 4.5–5.0) for vaccine-preventable conditions. The MH group had increased rates for all PPH conditions except *eclampsia* and *rheumatic heart disease*, which were too infrequent for reliable standardisation. Amongst chronic conditions, the highest number of admissions and highest relative risks were for respiratory conditions (AIRR 4.9, 95% CI 4.7–5.1), particularly *chronic obstructive airways disease* (AIRR 5.8, 95% CI 5.5–6.0). Rates were also significantly increased for metabolic conditions (AIRR 3.3, 95% CI 3.2–3.5) including *diabetic complications* (AIRR 5.4, 95% CI 5.1–5.8) and for cardiovascular conditions (AIRR 2.6, 95% CI 2.5–2.7). Amongst acute conditions, the highest rate ratios were for *seizures and epilepsy* (AIRR 7.4, 95% CI 7.0–7.8), which comprised 12% of total PPH admissions in the MH group but only 5% in the non-MH group.

**Table 2.** Episodes of Potentially Preventable Hospitalisation (PPH) in NSW, 2016–2017, comparing mental health service users (*n* = 178 009) with other NSW residents (*n* = 7 561 265) in the preceding year. PPH rate per 100 000 population, crude and adjusted incidence rate ratios (aIRR) after standardisation for (i) age group and (ii) age plus socioeconomic disadvantage

PPH condition	PPH episodes: *n* (%)		PPH rate per 100k		Incidence rate ratio		
	MH care	No MH care	MH care	No MH care	Unadjusted	aIRR (Age)	aIRR (Age and Disadvantage)
All	11 635	157 671	6536 (6418–6656)	2085 (2075–2096)	3.1 (3.1–3.2)	3.6 (3.6–3.7)	3.6 (3.5–3.6)
Chronic – all causes	5458	75 557	3066 (2985–3149)	999 (992–1006)	3.1 (3.0–3.2)	3.7 (3.6–3.8)	3.7 (3.6–3.7)
Chronic – respiratory	2521	28 021	1416 (1361–1473)	371 (366–375)	3.8 (3.7–4.0)	4.9 (4.7–5.1)	4.9 (4.7–5.1)
Chronic obstructive airways disease	1842	18 274	1035 (988–1083)	242 (238–245)	4.3 (4.1–4.5)	5.8 (5.6–6.1)	5.8 (5.5–6.0)
Asthma	588	8132	330 (304–358)	108 (105–110)	3.1 (2.8–3.3)	3.2 (2.9–3.5)	3.3 (3.0–3.6)
Bronchiectasis	91	1615	51 (41–63)	21 (20–22)	2.4 (1.9–3.0)	2.6 (2.1–3.2)	2.7 (2.2–3.3)
Chronic – cardiovascular	1331	26 550	748 (708–789)	351 (347–355)	2.1 (2.0–2.3)	2.7 (2.5–2.8)	2.6 (2.5–2.7)
Congestive cardiac failure	807	14 644	453 (423–486)	194 (191–197)	2.3 (2.2–2.5)	2.9 (2.7–3.1)	2.9 (2.7–3.1)
Angina	381	8725	214 (193–237)	115 (113–118)	1.9 (1.7–2.1)	2.3 (2.1–2.6)	2.3 (2.1–2.5)
Hypertension	127	2506	71 (59–85)	33 (32–34)	2.2 (1.8–2.6)	2.8 (2.4–3.3)	2.8 (2.3–3.3)
Rheumatic heart disease	16	675	9 (5–15)	9 (8–10)	1.0 (0.6–1.6)	–	–
Chronic – metabolic	1606	20986	902 (859–947)	278 (274–281)	3.3 (3.1–3.4)	3.4 (3.3–3.6)	3.3 (3.2–3.5)
Diabetes complications	1117	8639	627 (591–665)	114 (112–117)	5.5 (5.2–5.8)	5.6 (5.3–6.0)	5.4 (5.1–5.8)
Iron deficiency anaemia	448	12 206	252 (229–276)	161 (159–164)	1.6 (1.4–1.7)	1.8 (1.6–1.9)	1.7 (1.6–1.9)
Nutritional deficiency	41	141	23 (17–31)	2 (2–2)	12.4 (8.5–17.6)	14.2 (10.0–19.8)	15.1 (10.7–21.0)
Acute – all causes	5097	70 574	2863 (2785–2943)	933 (926–940)	3.1 (3.0–3.2)	3.4 (3.3–3.5)	3.3 (3.2–3.4)
Acute – infectious	3438	59 188	1931 (1867–1997)	783 (776–789)	2.5 (2.4–2.6)	2.8 (2.7–2.9)	2.7 (2.7–2.8)
Urinary tract infection	1151	16 824	647 (610–685)	223 (219–226)	2.9 (2.7–3.1)	3.3 (3.1–3.5)	3.3 (3.1–3.5)
Cellulitis	1175	15 031	660 (623–699)	199 (196–202)	3.3 (3.1–3.5)	3.5 (3.3–3.7)	3.4 (3.2–3.6)
Dental infection	564	14 854	317 (291–344)	196 (193–200)	1.6 (1.5–1.8)	1.9 (1.8–2.1)	2.0 (1.9–2.2)
Upper respiratory infection	427	10 985	240 (218–264)	145 (143–148)	1.7 (1.5–1.8)	2.0 (1.8–2.2)	1.9 (1.8–2.1)
Pneumonia	33	632	19 (13–26)	8 (8–9)	2.2 (1.5–3.1)	2.6 (1.8–3.6)	2.5 (1.7–3.5)
Pelvic inflammatory disease	88	862	49 (40–61)	11 (11–12)	4.3 (3.4–5.4)	3.3 (2.5–4.2)	3.3 (2.5–4.2)
Seizures and epilepsy	1418	8223	797 (756–839)	109 (106–111)	7.3 (6.9–7.8)	7.5 (7.1–7.9)	7.4 (7.0–7.8)
Acute – Other	250	3283	140 (124–159)	43 (42–45)	3.2 (2.8–3.7)	3.8 (3.4–4.3)	3.8 (3.3–4.2)
Gangrene	159	1914	89 (76–104)	25 (24–26)	3.5 (3.0–4.2)	4.2 (3.6–4.8)	4.2 (3.6–4.9)
Perforated ulcer	90	1357	51 (41–62)	18 (17–19)	2.8 (2.3–3.5)	3.3 (2.6–4.0)	3.1 (2.5–3.8)
Eclampsia	1	12	1 (0–3)	0 (0–0)	3.5 (0.1–23.9)	–	–
Vaccine preventable –all causes	1251	13 279	703 (664–743)	176 (173–179)	4.0 (3.8–4.2)	4.7 (4.4–4.9)	4.7 (4.5–5.0)
Vaccine-preventable pneumonia	501	7506	281 (257–307)	99 (97–102)	2.8 (2.6–3.1)	3.6 (3.3–3.9)	3.7 (3.4–4.0)
Other vaccine preventable	763	5839	429 (399–460)	77 (75–79)	5.6 (5.1–6.0)	6.1 (5.6–6.5)	6.0 (5.6–6.5)

*MH = mental health. PPH = Potentially Preventable Hospitalisations. Standardised rates are not calculated where event count is less than 30 in the study population (Rheumatic heart disease, Eclampsia).

PPH risk was increased for both categories of vaccine-preventable conditions. Risk was elevated for *other vaccine-preventable conditions* (AIRR 6.0, 95% CI 5.6–6.5), which includes hepatitis B and C, mumps, herpes zoster, diphtheria and pertussis, as well as for *vaccine-preventable pneumonia and influenza* (AIRR 3.7, 95% CI 3.4–4.0) which includes influenza and haemophilus or streptococcal pneumonia.

**Fig. 1.**
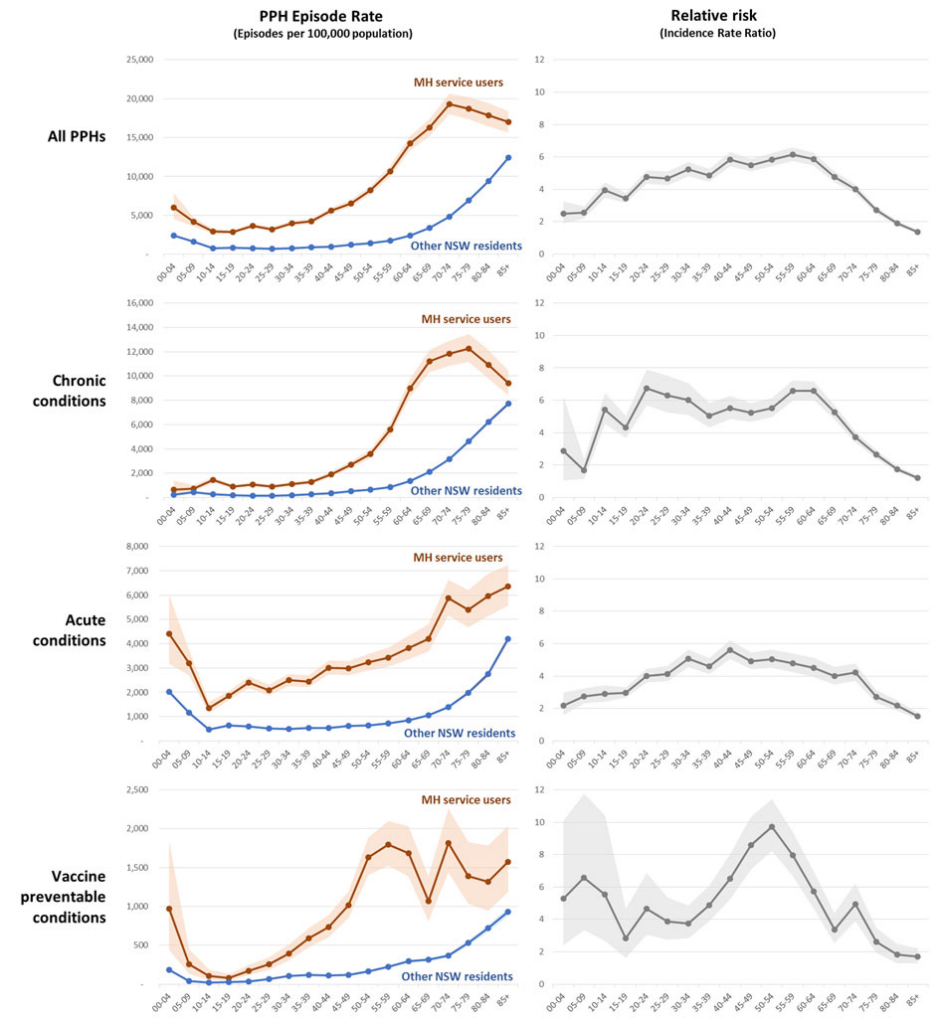
Rate of Potentially Preventable Hospitalisations (PPH) by age group, showing population risk (rate per 100,000 population) and relative risk (Incidence Rate Ratio). Shaded areas represent 95% confidence intervals.

The risk of PPH admission varied with age (Fig. 1, Supplementary Table s3). MH service users had an increased risk in each age band. PPH occurred at an earlier age in the MH group: MH service users aged 30–64 had a more than five-fold increase in the incidence rate of PPH admissions. The lowest PPH rate in MH service users was among 15–19-year olds (2837 episodes per 100 000 people, 95% CI 2617–3071) a rate that was only exceeded in the general population after the age of 65. The AIRR for PPH due to chronic conditions exceeded 5 from age 20 to 64 and admission rates increased steeply in MH service users aged in their 50s and 60s. Vaccine-preventable hospitalisations increased steeply in MH service users from age 35 onwards. The relative risk of PPH admission declined in MH service users aged over 80, due to absolute reduction in PPH admissions for chronic conditions and an increasing population baseline.

MH service users had more than five times the number of hospital days per 100 000 population (AIRR 5.2, 95% CI 5.2–5.3) compared with other NSW residents (Table 3). *Chronic obstructive pulmonary disease* was the largest cause of PPH hospital days in MH service users (10 921 days, 17% of total PPH Days). Preventable hospital days were increased in most conditions, particularly *chronic respiratory conditions* (ARR 6.1, 95% CI 6.0–6.2), *diabetes complications* (ARR 6.3, 95% CI 6.1–6.4), *vaccine-preventable conditions* (ARR 8.7, 95% CI 8.6–8.9), *acute pneumonia* (ARR 7.9, 95% CI 7.3–8.6) and *seizures and epilepsy* (ARR 10.3, 95% CI 10.0–10.6). The average length of stay was increased for almost all PPH conditions.

**Table 3.** Hospital days due to Potentially Preventable Hospitalisation (PPH) in NSW, 2016–2017, comparing people with and without community mental health (MH) care in the preceding year. PPH rate per 100 000 population, crude and adjusted rate ratios (ARR) after standardisation for (i) age group and (ii) age plus socioeconomic disadvantage

PPH condition	Length of stay (Days)		PPH days		PPH days per 100k		Incidence rate ratio		
	MH	No MH	MH	No MH	MH	No MH	Unadjusted	aIRR (Age)	aIRR (age and disadvantage)
All	5.4	3.9	63 134	615 534	35 467	8141	4.4 (4.3–4.4)	5.2 (5.2–5.3)	5.2 (5.2–5.3)
Chronic – all causes	5.3	4.2	29 020	317 847	16 303	4204	3.9 (3.8–3.9)	4.9 (4.8–4.9)	4.9 (4.8–4.9)
Chronic – respiratory	5.3	4.4	13 412	124 532	7534	1647	4.6 (4.5–4.7)	6.1 (6.0–6.2)	6.1 (6.0–6.2)
Chronic obstructive airways disease	5.9	5.3	10 921	97 642	6135	1291	4.8 (4.7–4.8)	6.6 (6.5–6.7)	6.6 (6.5–6.7)
Asthma	2.8	2.1	1664	17 474	935	231	4.0 (3.8–4.3)	4.4 (4.1–4.6)	4.5 (4.3–4.7)
Bronchiectasis	9.1	5.8	827	9416	465	125	3.7 (3.5–4.0)	3.8 (3.5–4.0)	4.1 (3.8–4.4)
Chronic – cardiovascular	5.7	4.7	7545	125 897	4239	1665	2.5 (2.5–2.6)	3.2 (3.1–3.3)	3.2 (3.1–3.2)
Congestive cardiac failure	7.5	6.5	6061	94 792	3405	1254	2.7 (2.6–2.8)	3.4 (3.4–3.5)	3.4 (3.3–3.5)
Angina	2.6	2.2	976	18 804	548	249	2.2 (2.1–2.4)	2.8 (2.7–3.0)	2.7 (2.6–2.9)
Hypertension	2.9	2.5	363	6231	204	82	2.5 (2.2–2.8)	3.1 (2.9–3.5)	3.1 (2.8–3.4)
Rheumatic heart disease	9.1	9.0	145	6070	81	80	1.0 (0.9–1.2)	0.9 (0.8–1.1)	0.8 (0.7–1.0)
Chronic – metabolic	4.5	3.1	7301	65 510	4101	866	4.7 (4.6–4.8)	5.4 (5.3–5.5)	5.3 (5.2–5.5)
Diabetes complications	5.6	5.5	6277	47 197	3526	624	5.6 (5.5–5.8)	6.4 (6.2–6.5)	6.3 (6.1–6.4)
Iron deficiency anaemia	2.3	1.5	1024	18 313	575	242	2.4 (2.2–2.5)	3.0 (2.8–3.1)	3.0 (2.8–3.1)
Nutritional deficiency	18.6	13.5	762	1908	428	25	17.0 (15.6–18.5)	17.2 (15.8–18.7)	19.4 (17.9–21.1)
Acute – all causes	4.3	3.3	21 799	230 863	12 246	3053	4.0 (4.0–4.1)	4.7 (4.6–4.7)	4.6 (4.6–4.7)
Acute – infectious	3.8	2.9	12 895	173 954	7244	2301	3.1 (3.1–3.2)	3.7 (3.6–3.7)	3.6 (3.5–3.7)
Urinary tract infection	4.3	3.6	4933	60 783	2771	804	3.4 (3.3–3.5)	4.1 (4.0–4.2)	4.1 (4.0–4.2)
Cellulitis	5.1	4.9	5965	74 050	3351	979	3.4 (3.3–3.5)	3.9 (3.8–4.0)	3.7 (3.7–3.8)
Dental infection	1.6	1.3	918	18 575	516	246	2.1 (2.0–2.2)	2.5 (2.4–2.7)	2.6 (2.4–2.7)
Upper respiratory infection	1.9	1.7	827	18 296	465	242	1.9 (1.8–2.1)	2.5 (2.4–2.7)	2.4 (2.2–2.5)
Pneumonia	19.0	6.4	628	4043	353	53	6.6 (6.1–7.2)	8.0 (7.4–8.6)	7.9 (7.3–8.6)
Pelvic inflammatory disease	2.9	2.6	252	2250	142	30	4.8 (4.2–5.4)	3.9 (3.4–4.5)	3.8 (3.3–4.4)
Seizures and epilepsy	3.5	2.8	5033	22 799	2827	302	9.4 (9.1–9.7)	10.1 (9.8–10.4)	10.3 (10.0–10.6)
Acute – Other	13.2	9.6	3296	31 464	1852	416	4.4 (4.3–4.6)	5.9 (5.7–6.0)	5.8 (5.6–5.9)
Gangrene	14.9	12.2	2375	23 262	1334	308	4.3 (4.2–4.5)	5.9 (5.7–6.1)	5.8 (5.6–6.0)
Perforated ulcer	10.2	6.0	918	8168	516	108	4.8 (4.5–5.1)	5.7 (5.4–6.1)	5.6 (5.3–6.0)
Vaccine preventable – all causes	11.2	6.0	13 979	80 262	7853	1061	7.4 (7.3–7.5)	8.4 (8.3–8.6)	8.7 (8.6–8.9)
Vaccine-preventable pneumonia	14.7	7.0	7387	52 484	4150	694	6.0 (5.8–6.1)	7.0 (6.9–7.2)	7.4 (7.3–7.6)
Other vaccine preventable	9.1	4.9	6953	28 365	3906	375	10.4 (10.1–10.7)	11.4 (11.1–11.7)	11.6 (11.3–11.9)

*MH = mental health. PPH = Potentially Preventable Hospitalisations. Eclampsia estimates suppressed due to low base rate.

**Fig. 2.**
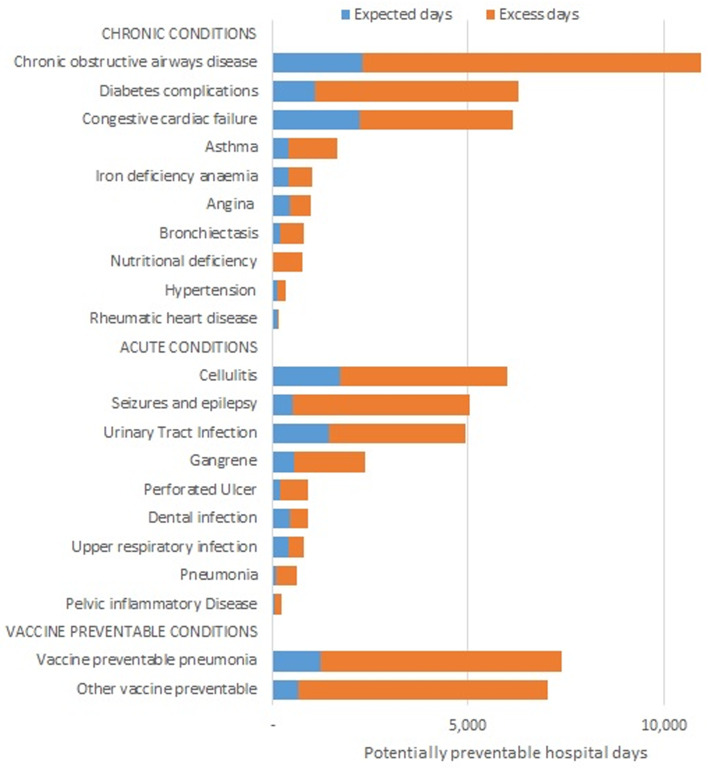
Excess potentially preventable hospital days in NSW mental health service users, 2016-17. Total observed days (63,134 days) compared with those expected if age-standardised admission rate and average length of stay for each condition matched those of other NSW residents (12,545 days).

MH service users experienced 63 134 days in the hospital due to PPH conditions in 2016–2017; 80% more than the expected number among other NSW residents (Fig. 2, Supplementary Table s4). The major contribution to excess bed days arose from *chronic obstructive pulmonary disease*, *diabetic complications*, *congestive cardiac failure*, *cellulitis*, *seizures* and *urinary tract infection*. *Vaccine-preventable conditions* contributed nearly one-quarter (24.5%) of excess bed days because of their greater incidence and substantially increased length of stay.

In sensitivity analyses (Table s5), excluding PPH admissions to private hospitals significantly increased the gap between MH service users and other NSW residents, because MH service users had fewer PPH admissions in private hospitals. Excluding PPH episodes occurring in specialised MH units, or PPH episodes occurring prior to a person's first community MH contact did not significantly alter the AIRR for PPH episode rates and caused a small but statistically significant reduction in AIRR for PPH days.

## Discussion

In the NSW population of nearly 8 million, MH service users had 3–4 times more preventable hospital admissions for physical health care, were admitted at a much younger age and spent substantially longer in hospital for equivalent conditions when compared to people without MH service contact. These findings are likely to reflect several interacting factors. First, rates of medical morbidity are increased in people with MH conditions. Our finding of more than five-fold increases in admission rates for chronic respiratory disease and diabetes complications is consistent with the increased prevalence of these conditions in people with MH conditions (Firth *et al*., 2019). However, we also found significantly increased admission rates for a wide range of medical conditions, consistent with evidence for broadly increased medical comorbidity across the spectrum of mental disorders (Momen *et al*., 2020). The large relative risk of admission for seizures and epilepsy has previously been reported (Mai *et al*., 2011; Kisely *et al*., 2015). There are shared risk factors and comorbidity between psychosis, depression and epilepsy (Patel *et al*., 2017), however, these hospitalisations may also reflect less effective care for seizure disorders or increased risk of medication-related seizures. Further study is needed to understand whether the large increase in hospitalisations for vaccine-related conditions in adults reflects increased rates of blood-borne viral infections, adult-onset conditions such as influenza, or conditions typically vaccinated against in childhood.

Second, increased preventable admission is likely to reflect health system factors. PPHs are an effective measure of access to primary and specialist care (Parchman and Culler, 1994; Basu *et al*., 2002; Gulliford, 2002; Gibson *et al*., 2013; Kim *et al*., 2019). Even where people with MH conditions have high rates of access to primary care (Saini *et al*., 2020), their engagement in effective care may be reduced due to negative individual experiences, service inflexibility or fragmentation, and broader social and financial barriers (Melamed *et al*., 2019).

We found that MH service users had five-fold increases in per capita rates of preventable hospital bed days. Of the total, 80% of bed days for MH service users were excess to those which would be expected if PPH incidence and the average length of stay were the same as for other NSW residents. MH service users are around 2% of the NSW population, but account for around 10% of preventable bed days for chronic respiratory and metabolic conditions and up to 20% of bed days for vaccine-preventable conditions. These findings highlight the significant burden of these preventable hospitalisations for individuals and for the health system. The finding of longer average stays for equivalent conditions underlines the importance of proactive consultation-liaison psychiatry, and integrated physical and MH care in general hospital settings (Oldham *et al*., 2019). The finding that MH service users were more likely to have repeated PPH admissions suggests opportunities for targeted intervention after a first PPH admission.

The current study examined whether increased PPH rates reported in previous longer-term studies were also observed over a 12-month time period more suitable for routine indicator reporting. Relative rates in the current study were *higher* than in earlier cohorts with longer follow-up periods. Kisely *et al*. (2015) examined people in their first 5 years of MH care, reporting up to two-fold increases in diabetes and angina admissions, but no increase for respiratory conditions, hypertension or congestive cardiac failure over 5-year follow up. Mai *et al*. (2011) followed MH service users over 16 years (to 2006) and reported two- to six-fold increases in PPH rates for a range of conditions. Higher relative risks in the current study may reflect time-dependent differences in risk. For example, we speculate that individuals may first access MH care at times of greater personal stress or instability, which may also be associated with greater risk of disrupted physical health care leading to hospitalisation.

The second aim of the current study was to examine the interaction between age and PPH in MH service users. One study has reported a U-shaped relationship between age and PPH rates, finding higher rates of PPH among children, youth and older people (Guo *et al*., 2001). We found a similar pattern in MH service users, but with higher levels at all ages and earlier onset of age-related increases. MH service users had increasing admission rates from around 40 years of age for vaccine-related conditions and around 50 years for chronic conditions. An alarming call to action was the finding that MH service users in their 20s already had higher rates of admission for chronic conditions than other NSW residents experienced until their mid-60 s. PPH admissions in younger MH service users are likely to reflect the earlier onset of acute and chronic medical conditions, and may provide a useful marker for identifying groups or individuals at greater risk.

### Limitations

This study uses linked data from routine health collections and therefore shares the limitations and biases of such data sets. In particular, the current dataset is biased towards the identification of people with more acute or severe MH conditions. Each year more than 10% of Australians access a MH service (Australian Institute of Health and Welfare, 2020), of whom approximately one-fifth, those with more severe and acute conditions, access the state government operated ‘public’ MH services included in this study. We do not have data on the approximately 8% of the Australian population whose only MH contact is with primary care, the federal government (Medicare) funded private specialist or non-government services. These people are therefore included in our non-MH reference population. Our reference population also included a small group of people who received specialist psychiatric admission but no community MH contact. This represents less than 5% of people admitted to hospital and less than 0.1% of the non-MH reference population.

Medical comorbidity and premature mortality in MH care is a global problem (Liu *et al*., 2017). An association between MH conditions and preventable hospital admissions has been demonstrated in diverse health care settings in the USA (Yoon *et al*., 2012; McGinty and Sridhara, 2014; Medford-Davis *et al*., 2018; Schmidt *et al*., 2018; Stockbridge *et al*., 2019), Denmark (Davydow *et al*., 2015, 2016), Taiwan (Lin *et al*., 2011) and Scotland (Payne *et al*., 2013). However, our specific findings may not generalise to the health systems of other countries. Australia has a federated health system, in which state governments provide hospital and community MH care, but the national government funds primary and outpatient specialist care. This creates a barrier to integration for people accessing (state) MH and (federal) physical health services. Different barriers are likely to exist in other health systems. We used Australia's national PPH definition, which is similar to that of the UK National Health Service (National Health Service Digital, 2019). Both definitions include more than 20 individual conditions grouped into acute, chronic and vaccine-preventable conditions, however, the NHS definition does not include rheumatic heart diseases, urinary tract infections, pyelonephritis or eclampsia. Both Australian and UK definitions are broader than definitions of Ambulatory Care Sensitive Conditions from the USA (11 conditions) and Canada (seven conditions) (Agency for Healthcare Research and Quality, 2018; Canadian Institute for Health Information, 2019). These differences in definition can have a significant impact on prevalence estimates (Purdy *et al*., 2009; Frick *et al*., 2017). We found that MH service users had increased rates for nearly all PPH conditions, which suggests that the inclusion or exclusion of individual conditions may have less impact on estimates of *relative* risk. However, our findings may not generalise directly to other health systems which use different definitions.

Socioeconomic disadvantage influences population PPH rates (Pappas *et al*., 1997; Ricketts *et al*., 2001; Gibbons *et al*., 2012), however, we found that increased PPH rates in MH service users were not accounted for by socioeconomic disadvantage. We used a regional measure of disadvantage, based on the person's area of residence, rather than individual measures of income, education or other personal characteristics. This approach was taken because (i) the datasets used do not contain detailed individual data for the MH or comparison populations, (ii) regional population data was required for standardisation and (iii) neighbourhood or regional indices of disadvantage are widely used in studies of this type. We used a validated Australian index (Australian Bureau of Statistics, 2006) which includes similar domains to those used elsewhere, such as the English Indices of Deprivation (McLennan *et al*., 2019). However, our negative findings may reflect the insensitivity of this approach if the social circumstances of an individual are not aligned with those of their broader region.

## Conclusion

Twelve-month PPH rates appear to be a potentially useful indicator for measuring the personal and health system impacts of increased medical morbidity in MH service users. Collaborative efforts such as the ‘Equally Well’ initiatives in the UK, Australia and New Zealand (National Mental Health Commission, 2016) highlight the complex health system changes needed to address these preventable conditions. In the long term, addressing these conditions may help to impact the problem of premature mortality in people with MH conditions, however, there is no current evidence directly linking PPH admissions to later mortality. The findings have potential clinical implications, highlighting the importance of somatic screening and integrated physical and MH care, and the need for awareness of earlier onset of somatic illness in MH consumers. Health services should consider routine screening for vaccination status and strategies for improving vaccination uptake for common conditions including hepatitis and influenza. The current study has not examined COVID-related admissions, however, our finding that MH service users had high admission rates for vaccine-preventable respiratory conditions underlines the importance of prioritising this group for COVID-19 vaccination as it becomes available (De Hert *et al*., 2020).

## Data Availability

No data are available. Access to NSW Health data is available to researchers only with the specific approval of the NSW Population and Health Services Research Ethics Committee (www.cancer.nsw.gov.au/research-and-data/nsw-population-health-services-research-ethics-com). That approval does not permit sharing of unit record data with other researchers.

## Supplementary material

For supplementary material accompanying this paper visit http://dx.doi.org/10.1017/S204579602100007X. click here to view supplementary material
