# Supplementary material for: Potentially preventable hospitalisations for physical health conditions in community mental health service users: a population-wide linkage study
Source: Epidemiol Psychiatr Sci. 2021 Mar 10;30:e22. doi: 10.1017/S204579602100007X (PMC8061153; doi:10.1017/S204579602100007X)
Supplement: Supplementary file 1 [file S204579602100007Xsup001.docx]

# Potentially preventable hospitalisations in mental health service users: a population-wide cohort study

# SUPPLEMENTARY MATERIAL

- - S1 Definition of Potentially Preventable Hospitalisations
  - S2 Details of cohort
  - S3 Supplementary data table for Figure 1 (Age x condition type interaction)
  - S4 Supplementary data table for Figure 2
  - S5 Sensitivity analysis: main results
  - S6 Index of Relative Socioeconomic Disadvantage (IRSD) additional information

**Table S1: Definition of PPH episodes.**

Specification is based on the Australian National Healthcare Agreement: PI 18-Selected potentially preventable hospitalisations, 2017. <https://meteor.aihw.gov.au/content/index.phtml/itemId/658499>

Diagnosis codes used The International Statistical Classification of Diseases and Related Health Problems Version 10, the National Centre for Classification in Health, Australia; AM - Australian Modification (ICD10-AM). ‘In any diagnosis’ uses 51 diagnosis recorded in up to 51 additional diagnosis fields per episode of care. Table adapted from Healthstats NSW <http://www.healthstats.nsw.gov.au/indicator/bod_acshos>.

| Category | ICD-10-AM codes | Comments |
| --- | --- | --- |
| Vaccine preventable conditions | | |
| Pneumonia and influenza (vaccine-preventable) | J10, J11, J13, J14 | In any diagnosis. Exclude people under 2 months. Rehabilitation records are excluded. |
| Other vaccine-preventable conditions | A08.0, A35, A36, A37, A80, B01, B05, B06, B16.1, B16.9, B18.0, B18.1, B26, G00.0 | In any diagnosis. Rotaviral Enteritis (A08.0) included for records with separation date 1 July 2007 onwards. Rehabilitation records are excluded. |
| Chronic conditions | | |
| Chronic Asthma | J45, J46 | As principal diagnosis. Exclude children aged less than 4 years. Rehabilitation records are excluded. |
| Congestive cardiac failure | I50, I11.0, J81 | As principal diagnosis. Exclude cases with the following cardiac procedure codes: Blocks 600-606, 608-650, 653-657, 660-664, 666, 669-682, 684-691, 693, 705-707, 717 and codes 33172-00[715], 33827-01[733], 34800-00[726], 35412-00[11], 38721-01[733], 90217-02[734], 90215-02[732]. Rehabilitation records are excluded. |
| Diabetes complications | E10, E11, E13, E14 | As principal diagnosis. Rehabilitation records are excluded. |
| COPD | J20, J41, J42, J43, J44 | J41-J44 as principal diagnosis. J20 as principal diagnosis with additional diagnoses of J41, J42, J43, J44. Rehabilitation records are excluded. |
| Bronchiectasis | J47, J20 | As principal diagnosis. J20 only with additional diagnosis of J47. Rehabilitation records are excluded. |
| Angina | I20, I24.0, I24.8, I24.9 | As principal diagnosis. Exclude cases according to the list of procedures excluded from the Congestive cardiac failure category above. Rehabilitation records are excluded. |
| Iron deficiency anaemia | D50.1, D50.8, D50.9 | As principal diagnosis. Rehabilitation records are excluded. |
| Hypertension | I10, I11.9 | As principal diagnosis.Exclude cases with procedure codes according to the list of procedures excluded from the Congestive cardiac failure category above. Rehabilitation records are excluded. |
| Nutritional deficiencies | E40, E41, E42, E43, E55.0, E64.3 | As principal diagnosis. Rehabilitation records are excluded. |
| Rheumatic heart diseases | I00, I01, I02, I05, I06, I07, I08, I09 | As principal diagnosis. Rehabilitation records are excluded. |
| Acute Conditions | | |
| Pneumonia (not vaccine-preventable) | J15.3, J15.4, J15.7, J16.0 | In any diagnosis. Exclude people under 2 months. Rehabilitation records are excluded. |
| Urinary tract infections, including pyelonephritis | N10, N11, N12, N13.6, N15.1, N15.9, N28.9, N39.0, N39.9 | As principal diagnosis. Rehabilitation records are excluded. |
| Perforated/bleeding ulcer | K25.0, K25.1, K25.2, K25.4, K25.5, K25.6, K26.0, K26.1, K26.2, K26.4, K26.5, K26.6, K27.0, K27.1, K27.2, K27.4, K27.5, K27.6, K28.0, K28.1, K28.2, K28.4, K28.5, K28.6 | As principal diagnosis. Rehabilitation records are excluded. |
| Cellulitis | L02, L03, L04, L08, L88, L98.0, L98.3 | As principal diagnosis. Exclude cases with any procedure except those in blocks 1820 to 2016, or if procedure is 30216-00, 30216-01, 30216-02, 30676-00, 30223-01, 30223-02, 30064-00, 90660-00, 90661-00, and this is the only listed procedure. Rehabilitation records are excluded. |
| Pelvic inflammatory disease | N70, N73, N74 | As principal diagnosis. Rehabilitation records are excluded. |
| Ear, nose and throat infections | H66, J02, J03, J06, J31.2 | As principal diagnosis. Rehabilitation records are excluded. |
| Dental conditions | K02, K03, K04, K05, K06, K08, K09.8, K09.9, K12, K13, K14.0 | As principal diagnosis. Rehabilitation records are excluded. |
| Convulsions and epilepsy | G40, G41, R56 | As principal diagnosis. Rehabilitation records are excluded. |
| Eclampsia | O15 | As principal diagnosis. Rehabilitation records are excluded. |
| Gangrene | R02, I70.24, E09.52 | R02 in any diagnosis. I70.2 and E09.52 as principal diagnosis. Rehabilitation records are excluded. |

**Table S2: Age and sex of people using NSW specialised mental health services in 2015-16 and 2016-17, compared to NSW population.**

|  | MH service use | Other NSW residents |
| --- | --- | --- |
| People | 178,009 (100.0%) | 7,561,265 (100.0%) |
|  |  |  |
| Sex |  |  |
| Male | 89,222 (50.1%) | 3,745,192 (49.5%) |
| Female | 88,689 (49.8%) | 3,816,171 (50.5%) |
| Other or unknown | 98 (0.1%) |  |
| Age Group |  |  |
| 00-04 | 1,153 (0.6%) | 499,817 (6.6%) |
| 05-09 | 4,762 (2.7%) | 493,476 (6.5%) |
| 10-14 | 11,337 (6.4%) | 442,413 (5.9%) |
| 15-19 | 21,357 (12.0%) | 444,177 (5.9%) |
| 20-24 | 16,881 (9.5%) | 513,355 (6.8%) |
| 25-29 | 16,411 (9.2%) | 559,549 (7.4%) |
| 30-34 | 15,682 (8.8%) | 555,213 (7.3%) |
| 35-39 | 15,843 (8.9%) | 499,497 (6.6%) |
| 40-44 | 14,641 (8.2%) | 502,978 (6.7%) |
| 45-49 | 13,631 (7.7%) | 486,521 (6.4%) |
| 50-54 | 11,183 (6.3%) | 482,961 (6.4%) |
| 55-59 | 8,733 (4.9%) | 468,265 (6.2%) |
| 60-64 | 6,438 (3.6%) | 415,739 (5.5%) |
| 65-69 | 5,399 (3.0%) | 381,127 (5.0%) |
| 70-74 | 4,281 (2.4%) | 289,214 (3.8%) |
| 75-79 | 3,703 (2.1%) | 213,350 (2.8%) |
| 80-84 | 3,114 (1.7%) | 151,008 (2.0%) |
| 85+ | 3,460 (1.9%) | 162,605 (2.2%) |

**Table s3: PPH rates by condition type and age group (Data for Figure 1)**

|  | **Any PPH** | | |  | **Chronic conditions** | | |
| --- | --- | --- | --- | --- | --- | --- | --- |
| **Age group** | **MH** | **No MH** | **IRR** |  | **MH** | **No MH** | **IRR** |
| 00-04 | 6,034 (4,558-7,836) | 2,412 (2,369-2,455) | 2.5 (1.9-3.3) |  | 647 (237-1,407) | 225 (212-238) | 2.9 (1.1-6.3) |
| 05-09 | 4,144 (3,579-4,774) | 1,621 (1,586-1,657) | 2.6 (2.2-3.0) |  | 734 (508-1,026) | 440 (422-459) | 1.7 (1.2-2.3) |
| 10-14 | 2,894 (2,575-3,242) | 734 (710-760) | 3.9 (3.5-4.4) |  | 1,437 (1,215-1,689) | 265 (250-280) | 5.4 (4.5-6.4) |
| 15-19 | 2,837 (2,617-3,071) | 820 (795-847) | 3.5 (3.2-3.8) |  | 897 (775-1,032) | 208 (195-222) | 4.3 (3.7-5.0) |
| 20-24 | 3,660 (3,380-3,957) | 771 (747-795) | 4.7 (4.4-5.2) |  | 1,081 (931-1,248) | 161 (150-172) | 6.7 (5.7-7.9) |
| 25-29 | 3,213 (2,945-3,499) | 689 (668-711) | 4.7 (4.2-5.1) |  | 893 (754-1,049) | 142 (132-152) | 6.3 (5.2-7.5) |
| 30-34 | 3,983 (3,678-4,306) | 760 (738-783) | 5.2 (4.8-5.7) |  | 1,128 (969-1,306) | 188 (176-199) | 6.0 (5.1-7.0) |
| 35-39 | 4,233 (3,920-4,564) | 872 (847-898) | 4.9 (4.5-5.3) |  | 1,290 (1,120-1,479) | 256 (242-271) | 5.0 (4.3-5.8) |
| 40-44 | 5,612 (5,234-6,011) | 960 (933-987) | 5.8 (5.4-6.3) |  | 1,926 (1,707-2,165) | 349 (333-366) | 5.5 (4.8-6.3) |
| 45-49 | 6,558 (6,138-7,000) | 1,195 (1,165-1,226) | 5.5 (5.1-5.9) |  | 2,703 (2,436-2,992) | 518 (498-538) | 5.2 (4.7-5.8) |
| 50-54 | 8,212 (7,690-8,761) | 1,410 (1,377-1,444) | 5.8 (5.4-6.2) |  | 3,601 (3,258-3,970) | 652 (629-675) | 5.5 (5.0-6.1) |
| 55-59 | 10,683 (10,011-11,388) | 1,734 (1,697-1,772) | 6.2 (5.8-6.6) |  | 5,614 (5,130-6,132) | 854 (828-881) | 6.6 (6.0-7.2) |
| 60-64 | 14,229 (13,328-15,174) | 2,428 (2,381-2,475) | 5.9 (5.5-6.3) |  | 8,985 (8,272-9,743) | 1,365 (1,330-1,401) | 6.6 (6.0-7.2) |
| 65-69 | 16,269 (15,203-17,390) | 3,409 (3,351-3,468) | 4.8 (4.5-5.1) |  | 11,215 (10,334-12,152) | 2,135 (2,089-2,182) | 5.3 (4.8-5.7) |
| 70-74 | 19,288 (18,014-20,629) | 4,802 (4,724-4,881) | 4.0 (3.7-4.3) |  | 11,831 (10,838-12,891) | 3,172 (3,108-3,237) | 3.7 (3.4-4.1) |
| 75-79 | 18,740 (17,364-20,195) | 6,905 (6,796-7,016) | 2.7 (2.5-2.9) |  | 12,275 (11,167-13,463) | 4,612 (4,522-4,704) | 2.7 (2.4-2.9) |
| 80-84 | 17,864 (16,411-19,411) | 9,393 (9,241-9,547) | 1.9 (1.7-2.1) |  | 10,904 (9,776-12,127) | 6,240 (6,115-6,367) | 1.7 (1.6-1.9) |
| 85+ | 16,974 (15,660-18,369) | 12,421 (12,252-12,591) | 1.4 (1.3-1.5) |  | 9,397 (8,426-10,449) | 7,720 (7,585-7,856) | 1.2 (1.1-1.4) |
|  | **Acute conditions** | | |  | **Vaccine preventable conditions** | | |
| **Age group** | **MH** | **No MH** | **IRR** |  | **MH** | **No MH** | **IRR** |
| 00-04 | 4,418 (3,171-5,994) | 2,018 (1,979-2,058) | 2.2 (1.6-3.0) |  | 970 (443-1,841) | 184 (172-196) | 5.3 (2.4-10.1) |
| 05-09 | 3,194 (2,701-3,753) | 1,162 (1,132-1,192) | 2.7 (2.3-3.2) |  | 259 (134-452) | 39 (34-45) | 6.6 (3.3-11.8) |
| 10-14 | 1,360 (1,144-1,605) | 471 (451-492) | 2.9 (2.4-3.4) |  | 107 (53-191) | 19 (15-24) | 5.5 (2.7-10.4) |
| 15-19 | 1,862 (1,685-2,053) | 627 (604-650) | 3.0 (2.7-3.3) |  | 83 (49-131) | 29 (25-35) | 2.8 (1.6-4.6) |
| 20-24 | 2,405 (2,179-2,648) | 600 (579-622) | 4.0 (3.6-4.4) |  | 174 (118-249) | 37 (32-43) | 4.7 (3.1-6.9) |
| 25-29 | 2,083 (1,869-2,316) | 504 (486-523) | 4.1 (3.7-4.6) |  | 255 (184-345) | 66 (59-73) | 3.9 (2.7-5.3) |
| 30-34 | 2,496 (2,256-2,754) | 493 (475-512) | 5.1 (4.5-5.6) |  | 397 (305-508) | 106 (98-115) | 3.7 (2.8-4.9) |
| 35-39 | 2,442 (2,206-2,697) | 531 (511-552) | 4.6 (4.1-5.1) |  | 589 (476-720) | 120 (111-130) | 4.9 (3.9-6.1) |
| 40-44 | 3,012 (2,737-3,308) | 537 (517-558) | 5.6 (5.1-6.2) |  | 736 (603-889) | 113 (104-123) | 6.5 (5.2-8.0) |
| 45-49 | 2,986 (2,704-3,288) | 607 (586-629) | 4.9 (4.4-5.5) |  | 1,015 (853-1,197) | 118 (109-128) | 8.6 (7.1-10.3) |
| 50-54 | 3,226 (2,902-3,576) | 639 (617-662) | 5.0 (4.5-5.6) |  | 1,635 (1,407-1,890) | 168 (157-180) | 9.7 (8.2-11.4) |
| 55-59 | 3,432 (3,056-3,842) | 716 (692-741) | 4.8 (4.2-5.4) |  | 1,796 (1,527-2,099) | 226 (212-240) | 8.0 (6.7-9.4) |
| 60-64 | 3,818 (3,358-4,323) | 846 (819-875) | 4.5 (4.0-5.1) |  | 1,687 (1,386-2,033) | 294 (278-311) | 5.7 (4.7-7.0) |
| 65-69 | 4,208 (3,675-4,797) | 1,055 (1,022-1,088) | 4.0 (3.5-4.6) |  | 1,071 (811-1,387) | 318 (300-336) | 3.4 (2.5-4.4) |
| 70-74 | 5,870 (5,177-6,631) | 1,395 (1,353-1,438) | 4.2 (3.7-4.8) |  | 1,813 (1,438-2,257) | 367 (345-389) | 4.9 (3.9-6.2) |
| 75-79 | 5,401 (4,675-6,208) | 1,983 (1,924-2,043) | 2.7 (2.4-3.1) |  | 1,391 (1,036-1,829) | 529 (499-561) | 2.6 (1.9-3.5) |
| 80-84 | 5,965 (5,139-6,887) | 2,752 (2,670-2,837) | 2.2 (1.9-2.5) |  | 1,315 (944-1,784) | 721 (679-765) | 1.8 (1.3-2.5) |
| 85+ | 6,365 (5,571-7,241) | 4,203 (4,104-4,304) | 1.5 (1.3-1.7) |  | 1,571 (1,190-2,035) | 928 (882-976) | 1.7 (1.3-2.2) |

**Table s4: Data for Figure 2.** **Expected vs observed PPH hospital bed days for NSW MH service users.**

Expected days if condition-specific incidence rate and length of stay matched other NSW residents. One hospitalisation may include more than one PPH diagnosis, so the total of condition-specific days may exceed the subgroup or overall total.

|  | Expected PPH bed days | Excess PPH bed days | Total PPH bed days |
| --- | --- | --- | --- |
| All | 14,524 | 48,975 | 63,499 |
| CHRONIC | 7,490 | 21,665 | 29,155 |
| Chronic obstructive airways disease | 2,300 | 8,649 | 10,949 |
| Diabetes complications | 1,113 | 5,179 | 6,292 |
| Congestive cardiac failure | 2,233 | 3,912 | 6,145 |
| Asthma | 413 | 1,255 | 1,668 |
| Iron deficiency anaemia | 431 | 594 | 1,025 |
| Angina | 444 | 535 | 979 |
| Bronchiectasis | 222 | 605 | 827 |
| Nutritional deficiency | 45 | 717 | 762 |
| Hypertension | 147 | 216 | 363 |
| Rheumatic heart disease | 145 | 0 | 145 |
| ACUTE | 5,449 | 16,462 | 21,911 |
| Cellulitis | 1,748 | 4,276 | 6,024 |
| Seizures and epilepsy | 540 | 4,524 | 5,064 |
| Urinary Tract Infection | 1,435 | 3,514 | 4,949 |
| Gangrene | 548 | 1,827 | 2,375 |
| Perforated Ulcer | 193 | 728 | 921 |
| Dental infection | 438 | 481 | 919 |
| Upper respiratory infection | 433 | 396 | 829 |
| Pneumonia | 95 | 533 | 628 |
| Pelvic inflammatory Disease | 53 | 199 | 252 |
| VACCINE PREVENTABLE | 1,896 | 12,204 | 14,100 |
| Vaccine preventable pneumonia | 1,242 | 6,163 | 7,405 |
| Other vaccine preventable | 672 | 6,384 | 7,056 |

**Table s5: Sensitivity analyses. Main findings.**

Age standardised rates of PPH admissions and PPH days per 1000 person years after excluding: (1) PPH admissions to private hospitals; (2) PPH episodes whose whole duration occurred in a designated mental health unit; (3) people whose only community mental health contact occurred at the same time as a hospital episode, and; (4) PPH episodes commencing before the person’s first recorded community mental health contact within the observation period.

|  | Age standardised PPH rates per 1000 population per annum (Rate, 95% CI) | | | | |
| --- | --- | --- | --- | --- | --- |
|  | **MH service users** |  | **Other NSW residents** |  | **IRR** |
| **PPH Episodes** |  |  |  |  |  |
| All Data | 7,542 (7,415-7,671) |  | 2,085 (2,075-2,096) |  | 3.62 (3.55-3.68) |
| 1. Excluding private hospitals | 7,117 (6,994-7,242) |  | 1,712 (1,702-1,721) |  | 4.16 (4.08-4.23)* |
| 2. Excluding mental health episodes | 7,460 (7,333-7,588) |  | 2,084 (2,074-2,095) |  | 3.58 (3.52-3.64) |
| 3. Excluding brief MH contact during hospital care | 7,347 (7,222-7,474) |  | 2,085 (2,075-2,095) |  | 3.52 (3.46-3.59) |
| 4. Excluding PPH before first mental health contact | 7,564 (7,436-7,693) |  | 2,085 (2,075-2,096) |  | 3.63 (3.56-3.69) |
| **PPH Days** |  |  |  |  |  |
| All Data | 42,570 (42,267-42,874) |  | 8,141 (8,120-8,161) |  | 5.23 (5.19-5.27) |
| 1. Excluding private hospitals | 40,610 (40,314-40,907) |  | 7,026 (7,007-7,045) |  | 5.78 (5.74-5.83)* |
| 2. Excluding mental health episodes | 40,822 (40,526-41,120) |  | 8,129 (8,109-8,149) |  | 5.02 (4.98-5.06)* |
| 3. Excluding brief MH contact during hospital care | 39,473 (39,181-39,766) |  | 8,135 (8,115-8,156) |  | 4.85 (4.81-4.89)* |
| 4. Excluding PPH before first mental health contact | 40,533 (40,238-40,830) |  | 8,141 (8,120-8,161) |  | 4.98 (4.94-5.02)* |

Note * Sensitivity analysis shows significantly different estimate compared to main analysis

**Table s6: Australian Bureau of Statistics Index of Relative Socioeconomic Disadvantage (IRSD) additional information**

The Australian Bureau of Statistics Index of Relative Socioeconomic Disadvantage (IRSD) is an index score calculated for each Australian geographical area by combining 17 census-derived variables related to income, government welfare support, education, home ownership, employment, household structure and English language proficiency

- % Occupied private dwellings with no internet connection
- % Employed people classified as Labourers
- % People aged 15 years and over with no post-school qualifications
- % People with stated annual household equivalised income between $13,000 and $20,799 (approx. 2nd and 3rd deciles)
- % Households renting from Government or Community organisation
- % People (in the labour force) unemployed
- % One parent families with dependent offspring only
- % Households paying rent less than $120 per week (excluding $0 per week)
- % People aged under 70 who have a long-term health condition or disability and need assistance with core activities
- % Occupied private dwellings with no car
- % People who identified themselves as being of Aboriginal and/or Torres Straight Islander origin
- % Occupied private dwellings requiring one or more extra bedrooms (based on Canadian National Occupancy Standard)
- % People aged 15 years and over who are separated or divorced
- % Employed people classified as Machinery Operators and Drivers
- % People aged 15 years and over who did not go to school
- % Employed people classified as Low Skill Community and Personal Service Workers
- % People who do not speak English well

Detailed information on its calculation can be found in 2039.0 - Information Paper: An Introduction to Socio-Economic Indexes for Areas (SEIFA), 2006 <https://www.abs.gov.au/ausstats/abs@.nsf/mf/2039.0>.
